# Antiepileptic drug carbamazepine promotes horizontal transfer of plasmid-borne multi-antibiotic resistance genes within and across bacterial genera

**DOI:** 10.1038/s41396-018-0275-x

**Published:** 2018-10-05

**Authors:** Yue Wang, Ji Lu, Likai Mao, Jie Li, Zhiguo Yuan, Philip L. Bond, Jianhua Guo

**Affiliations:** Advanced Water Management Centre, The University of Queensland, St. Lucia, Brisbane, QLD 4072 Australia

**Keywords:** Antibiotics, Transcriptomics, Proteomics

## Abstract

Antibiotic resistance is a severe global threat for public health, causing around 700,000 deaths per year. Horizontal gene transfer (HGT) is one of the most significant pathways to disseminate antibiotic resistance. It is commonly acknowledged that sub-minimum inhibition concentrations of antibiotics are major contributors in promoting antibiotic resistance through HGT. Pharmaceuticals are occurring in our environments at increased levels, yet little is known whether non-antibiotic pharmaceuticals cause or accelerate the dissemination of antibiotic resistance. Here, we report for the first time that the antiepileptic drug, carbamazepine, promotes conjugative transfer of antibiotic resistance genes. It was seen that environmentally relevant concentrations of carbamazepine (e.g., 0.05 mg/L) significantly enhanced the conjugative transfer of multiresistance genes carried by plasmid within and across bacterial genera. The underlying mechanisms of the enhanced HGT were revealed by detecting oxidative stress and cell membrane permeability, in combination with MinION DNA sequencing, genome-wide RNA sequencing, and proteomic analysis. Carbamazepine induced a series of acute responses, including increased levels of reactive oxygen species, the SOS response; increased cell membrane permeability, and pilus generation. Expressional levels of genes related to these processes were significantly upregulated during carbamazepine exposure. Given that HGT occurs widely among different species in various environments, these findings are an early warning for a wide assessment of the roles of non-antibiotic pharmaceuticals in the spread of antibiotic resistance.

## Introduction

Antimicrobial resistance (AMR), the ability of bacteria to resist the effects of antimicrobial agents, is the single most important infectious disease threat to human beings. As one of the biggest threats to global health defined by the World Health Organization, it is predicted that if no action is taken from now, as many as 10 million people will die as a result of AMR by 2050 [1]. The spread of antibiotic resistance is mainly attributed to selective pressure caused by extensive antibiotic consumption [2]. Horizontal gene transfer (HGT) is a major driver for disseminating antibiotic resistance genes (ARGs) in the environment [3]. Among the three pathways of HGT, conjugation, transformation, and transduction, conjugation is the most dominant routine [4]. Conjugation is the transfer of DNA between donor and recipient bacteria, which is accomplished by direct physical cell-to-cell contact through a pilin bridge generated by the donor cell [5, 6]. Generally, spontaneous frequency of conjugation is very low, however, antibiotics in the environment, especially at concentrations lower than the minimum inhibition concentration (MIC), will serve as selective drivers of the conjugation processes [7]. In addition, the conjugative transfer of DNA may occur within or across bacterial genera, although the latter may occur less frequently [6].

A common view is that antibiotic resistance may largely emerge from hospital environments and many previous studies have focused on the dissemination of antibiotic resistance in clinical settings. Nevertheless, there is increasing evidence that various natural or engineered ecosystems, and in particular wastewater treatment plants (WWTPs), are behaving as crucial reservoirs for antibiotic-resistant bacteria (ARB) and ARGs [8]. Surprisingly, it was recently documented that more ARB and ARGs are detected in residential areas as opposed to those detected in hospital wastewater [9]. In addition, mobile genetic elements containing ARGs, such as class 1 integrons, are highly prevalent in the environment, even in the absence of antibiotic selection [10]. As a consequence, there are concerns that non-antibiotic environmental contaminants present in wastewater are playing roles in the dissemination of ARGs. Recently, a few studies report that environmental contaminants, such as nanomaterials [11], disinfectants [12], disinfection by-products [13], and ionic liquids [14] have the potential to disseminate ARGs by promoting HGT. While these discoveries are disturbing, the underlying mechanisms of how these contaminants promote HGT has not been determined. Moreover, as one of the most commonly detected environmental contaminants, the potential roles of non-antibiotic pharmaceuticals in the dissemination of antibiotic resistance are largely neglected.

Non-antibiotic pharmaceuticals can enter environmental settings via human excretion and domestic sewage, husbandry manure, and hospital and manufacturing wastewater [15]. Among them, the commonly prescribed antiepileptic carbamazepine is often detected as one of the highest pharmaceutical residues in aquatic environments that include groundwater, surface water, wastewater, and drinking water [16]. The consumption of carbamazepine worldwide is as high as 1014 tons each year [16]. However, because of its resistance to biodegradation [17] and low adsorption onto sludge [18], carbamazepine can accumulate and remain persistent in water environments. Typically, less than 10% of the contaminant is removed during wastewater treatment [16]. As a result, there is a high possibility for carbamazepine and ARB to be present in aquatic environments. Therefore, we hypothesize that carbamazepine could play an antibiotic-like role by increasing the spread of ARGs in the environment. Currently, there is no study that reports whether the non-antibiotic pharmaceutical carbamazepine promotes HGT of ARGs.

In this study, we investigated whether carbamazepine is able to promote the conjugative transfer of antibiotic multiresistance genes carried by a plasmid within and across bacterial genera. We used two model systems, to study intragenera and intergenera transfer. Our results demonstrated that the antiepileptic drug carbamazepine could significantly enhance conjugative frequency in both intragenera and intergenera transfer. These findings were supported by phenotypic tests and through results of MinION plasmid sequencing, genome-wide RNA sequencing, and proteomic analysis. We revealed that carbamazepine induced a series of responses that included increased levels of reactive oxygen species (ROS), triggering of the SOS response, increased cell membrane permeability, and the increased generation of pilus. This is the first study to explore how a non-antibiotic pharmaceutical increases the horizontal transfer of plasmid-borne multiresistance genes. The findings change our understanding of the dissemination of antibiotic resistance enhanced by non-antibiotic pharmaceuticals and cause us to re-think the spread of ARGs in environment.

## Materials and methods

### Bacterial strains and culture media

*Escherichia coli* K-12 LE392 carrying the RP4 plasmid, with resistance genes of tetracycline (Tet), kanamycin (Ka), and ampicillin (Amp) was chosen as the donor. A mutant strain of *E. coli* K-12 MG1655 obtained from our previous study [19] and *Pseudomonas putida* KT2440, which are both resistant to chloramphenicol (Chl), were chosen as the recipients for the intragenera and intergenera transfers, respectively. Culture conditions for growth of the donor and recipients are described in Supporting Information (SI) Text 1.

### Determining MICs

MIC of donor and recipient strains towards antibiotics were determined as done previously [13, 20]. In detail, the bacteria were grown and diluted to approximately 10^5^ cfu/mL. To each well within the 96-well plates, 5 μL of the bacterial culture, 15 μL of antibiotics (with different concentrations), and 130 μL of fresh LB media were added. Ethanol or sterilized PBS were used as blank controls. This was required as the stock solutions of Chl and carbamazepine were prepared in ethanol, and the solutions of Tet, Ka, and Amp were prepared in sterilized milli-Q water. The plates were incubated at 30 °C for 20 h before the OD_600_ was measured on the plate reader (Tecan Infinite M200, Switzerland). MICs of the bacterial strains were determined as the concentration of antibiotic which inhibited 90% of the growth. Each bacterial strain under the inhibition of the different antibiotics was tested at least in triplicate.

### Conjugation experiments in the presence of carbamazepine

Conjugation experiments between donor and recipient bacteria were conducted in the presence of carbamazepine. Carbamazepine was added to the donor and recipient mixtures (total volume of 1 mL) that contained 10^8^ cfu/ mL of both the donor and recipient, to achieve final concentrations of 0.05, 0.5, 5.0, 10.0, 12.5, 25.0, and 50.0 mg/L. These concentrations of carbamazepine covered the environmental-relevant levels and the sub-MIC concentrations of the bacteria. The conjugation mating systems were then mixed by vortexing. Substrate free phosphate-buffered saline (PBS, pH = 7.2) was used in the mating systems to avoid providing growth conditions for the donor, recipient, or the transconjugant during the mating period. After incubation at 25 °C for 8 h without shaking, the mating systems were mixed and used to inoculate on LB agar selection plates (Sigma-Aldrich, USA) containing antibiotics. The plates were incubated at 30 °C for 48 h and then colonies of the transconjugants (recipients that received the RP4 plasmid) and recipients (not receiving the plasmid) were counted separately (media details of the selection plates are presented in SI Text 2). In addition, the same sets of mating system experiments were established with the addition of thiourea, an ROS scavenger (final concentration of 100 μM). The transfer frequency was calculated from the number of transconjugants detected divided by the total number of recipients. All the conjugation mating systems under the different levels of carbamazepine were performed at least as biological triplicates. In parallel, separate batches of both the donor and recipient bacteria were plated onto the transconjugant selection plates to rule out the occurrence of spontaneous mutation of the strains. In addition, to rule out any possible selective advantage that could potentially be caused by the RP4 plasmid under exposure of carbamazepine, growth curves of randomly selected transconjugants and the corresponding recipients were performed in biological triplicates (see SI, Text 3).

### Reverse conjugation experiments under the exposure of carbamazepine

Transconjugant bacteria obtained from the intergenera transfer experiment (*P. putida* KT2440 with RP4 plasmid) were used as the donor, and *E. coli* MG1655, with resistance to Chl, was used as the recipient. The conjugation mating systems were set up as described above containing the different concentrations of carbamazepine. The mating systems were then mixed by vortexing and incubated at 25 °C for 8 h without shaking. Following that the mating systems were mixed and used to inoculate on Difco^TM^ m Endo Agar selection plates, containing antibiotics, and then incubated as described above. Then colonies of the transconjugants and recipients were counted and the transfer frequencies were determined as described above.

### Measurement of ROS and cell membrane permeability

A CytoFLEX S flow cytometer (Beckman Coulter, USA) was applied for the detection of both ROS and cell membrane permeability. For the ROS detection, the DCFDA cellular ROS detection assay kit (abcam^®^, UK) was employed according to manufacturer’s instructions. Cell membrane permeability was tested using the propidium iodide dye (PI) at the concentration of 2 mM (Life Technologies, USA) using the previously described methods [14, 21] (see details in SI Text 4).

### Plasmid extraction, gel electrophoresis, and MinION sequencing

Five transconjugants from the transconjugant-selective plates were randomly selected, cultured in LB broth overnight and stored at −80 °C. Plasmid extractions for the selected transconjugants was performed using the Invitrogen^TM^ PureLink^®^ Quick Plasmid Miniprep Kit (Life Technologies, USA), following the manufacturer’s instructions. Following extraction, agarose gel electrophoresis was applied to verify the presence of plasmids in each transconjugant. MinIon Nanopore sequencing of the transconjugant plasmids was performed to confirm that the these contained the same genes as the original donor bacterial RP4 plasmid (see SI Text 5).

### RNA extraction, genome-wide RNA sequencing, and bioinformatics

The intergenera conjugation mating systems were established as described above, except the levels of carbamazepine were 0.0 mg/L, 0.05 mg/L, 10.0 mg/L, and 50.0 mg/L. These conditions were referred to as control, low-dosage, medium-dosage, and high-dosage, respectively. After a two-hour mating period the total RNA in each system was extracted using the RNeasy Mini Kit (QIAGEN^®^, Germany) following the manufacturer’s instructions with the addition of an extra bead-beating step for the cell lysis process. RNA samples were then submitted to Macrogen Co. (Seoul, Korea) for strand specific cDNA library construction and Illumina paired-end sequencing (HiSeq 2500, Illumina Inc., San Diego, CA). The bioinformatic analysis was performed based on the sequence data as detailed (SI Text 6).

### Protein extraction and proteomics analysis

Another set of intergenera mating systems were established as described above for protein extractions except the carbamazepine levels were 0.0 mg/L, 0.05 mg/L, 10.0 mg/L, and 50.0 mg/L. After 8 h mating, bacteria were harvested by centrifugation at 12,000× *g* for 10 min. Total bacterial protein was extracted from these cell pellets using the B-PER method, followed by reduction, alkylation, digestion, and ziptip clean-up procedures. This was performed as done previously [22]. Following this extraction, 5 μg aliquot of the purified protein from each triplicate sample were applied for mass spectrometry analysis and for construction of protein libraries by information dependent analysis (IDA). Then, another 1 μg aliquot of the purified protein from each triplicate sample were used for SWATH-MS analysis. Samples were applied to a Triple-T of 5600 instrument (ABSciex, USA) equipped with a Nanospray III interface, using the settings as described previously [23]. The ProteinPilot software (ABSciex, USA), PeakView v2.1 (ABSciex, USA), and R-based program MSstats [24] were applied for the data analysis (SI, Text 7).

### Statistical analysis

Data were expressed as mean ± SD. SPSS for Mac version 24.0 was applied for data analysis. Independent-sample *t* tests were performed, and *P* values were corrected using the “Benjamini–Hochberg” method [25] and presented as *P*_adj_. *P*_adj_-values less than 0.05 were considered to be statistically significant. All the experiments were conducted in triplicate.

### Data availability

All data was deposited in publicly accessible databases. Plasmid sequencing data are accessible through Sequence Read Archive of NCBI (SRP136301). RNA sequence data are accessible through Gene Expression Omnibus of NCBI (GSE112064). The mass spectrometry proteomics data have been deposited to the ProteomeXchange Consortium via the PRIDE [26] partner repository with the dataset identifier PXD009279.

## Results

### Carbamazepine significantly increases the conjugative transfer frequency

**Fig. 1:**
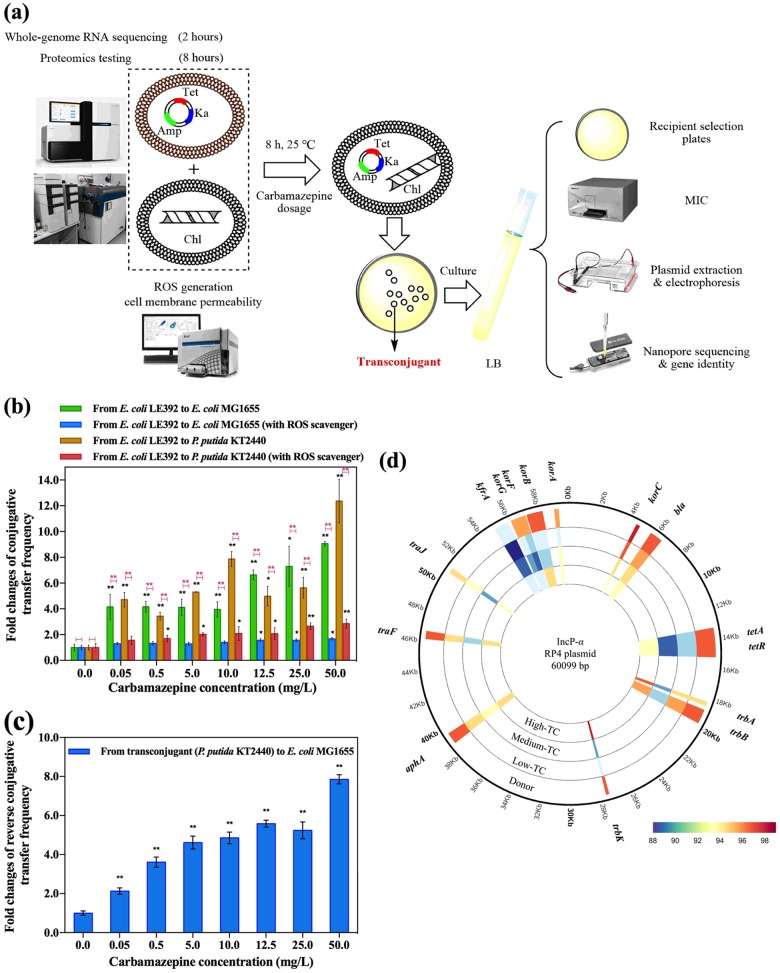
Phenotypic results for conjugative transfer of ARGs induced by different concentrations of carbamazepine. **a** Mating experimental design and methodologies. **b** Fold changes of conjugative transfer frequency. **c** Fold changes of reverse conjugative transfer frequency. **d** Identity similarities of key genes in the plasmids extracted from donor and transconjugants (low-TC, medium-TC, and high-TC refer to the plasmids from the transconjugants under the exposure of 0.05, 10.0, and 50.0 mg/L carbamazepine, respectively). Significant differences between carbamazepine-dosed samples and the control were detected using independent-sample *t* test, *P* values were corrected by the “Benjamini–Hochberg” method as *P*_adj_, *P_adj_ < 0.05, and **P_adj_ < 0.01

To test the effects of carbamazepine on conjugative transfer, we evaluated both the intragenera and intergenera transfer of the RP4 plasmid under the exposure of seven different sub-MIC concentrations of carbamazepine (in the range of 0.05–50 mg/L). These tests included the environmentally relevant concentrations of carbamazepine at between 0.05 and 0.5 mg/L. Following counting of the colony numbers on transconjugant-selective plates and recipient-selective plates, the transfer frequency was calculated by dividing the number of transconjugants counted by the number of recipients (Fig. 1a).

It was shown that for the seven concentrations of carbamazepine used here, that the intragenera transfer was significantly enhanced (*P*_adj_ < 0.05). Spontaneous transfer resulted in about 6.85 × 10^−5^ transconjugants per recipient, and the frequency increased with the dosage of carbamazepine (Fig. S1). Correspondingly, the fold changes of conjugative transfer frequency were significantly increased (*P*_adj_ < 0.01) (Fig. 1b). It was seen that at the low dosage used in this study (0.05 mg/L), the fold change was about four times, and this remained as such up to the 10.0 mg/L level of carbamazepine. From the 12.5 mg/L level and higher the fold changes increased with the increased carbamazepine concentration, reaching to be more than 9 times at the exposure of 50.0 mg/L.

In comparison to intragenera transfer, the intergenera transfer occurs between different bacterial genera, and the increased phylogenetic distance between these taxa may cause a lower frequency of of conjugation [6]. Likely this was the case, as much less spontaneous intergenera transconjugation occured in comparison to that in the intragenera transfer (a low count of 2.67 × 10^−6^ transconjugants per recipient was detected). Nevertheless, carbamazepine was also seen to increase the intergenera transfer significantly (*P*_adj_ < 0.05) (Fig. 1b and Fig. S1). Colony numbers on the transconjugant selection plates increased with increasing carbamazepine levels (Fig. S2). The corresponding transfer resulted in up to 4.31 × 10^−5^ transconjugants per recipient, which was more than 12 times higher than the spontaneous transfer (Fig. 1b). Lower dosages of carbamazepine (0.05, 0.5, and 5.0 mg/L) were also seen to significantly increase the intergenera transfer (*P*_adj_ < 0.01) by about a 4-fold enhancement.

In order to investigate whether the transconjugant is transferable, we performed the reverse conjugative experiment. In this event the RP4 plasmid is transferred from *P. putida* to *E. coli*. It was seen that the conjugative transfer frequency was again significantly enhanced in the presence of carbamazepine (*P*_adj_ < 0.01) (Fig. S3). Correspondingly, significant increases in the fold changes of the transfer frequency occurred in the presence of carbamazepine (*P*_adj_ < 0.01) (Fig. 1c). For example, at the dosage of 0.05 mg/L carbamazepine, the conjugative transfer frequency increased more than two times, and at 50.0 mg/L carbamazepine the fold change was as high as 7-fold.

We conducted various analyses to verify the conjugative plasmid transfer (Fig. 1a). For the initial intergenera transfer, transconjugant colonies were chosen and re-grown on *Pseudomonas* selection plates to verify the identity of the recipient cells. Secondly, the MICs of the transconjugants towards the four antibiotics, Tet, Ka, Amp, and Chl, were determined. The MIC measures the species sensitivity towards a particular antibiotic [27], and all the recipient transconjugants examined here exhibited the multiple resistance that was expected upon the successful transfer of the RP4 plasmid. The MICs of the transconjugants were at least the same or greater than those of the donor or recipients individually (Tables S1 and S2). Additionally, plasmids of the donor, recipient, and randomly selected transconjugants were extracted and examined by electrophoresis. All the selected transconjugants were seen to possess the plasmid of the same size to that of the donor (Fig. S4). To further verify the RP4 plasmid transfer, we applied MinION sequencing to demonstrate that the ARGs and transfer-related genes in the plasmids of transconjugants were the same as those in the donor bacterial strain. Based on comparative plasmid analyses, we found that the transconjugants’ plasmids, contained all the key genes of the RP4 plasmid. This included the genes *tetA*-*tetR*, *korB*, *trbB*, *bla*, *traF*, *traJ*, *trbA*, *korA*, and *trbK* [28–30], and these gene sequences matched well with those in the donor plasmid (Fig. 1d).

In order to rule out that plasmid RP4 confers a selective advantage to bacteria exposed to the pharmaceutical, growth curves of the randomly selected transconjugants and recipients were compared under different carbamazepine concentrations. In terms of the lag time and maximum growth rate between transconjugants and their corresponding recipient at the same carbamazepine concentration, RP4 plasmid did not show any significant selective advantage to the bacteria exposed to carbamazepine (Fig. S5, Tables S3-S7).

Consequently, it can be concluded these transconjugants are indeed the recipient bacteria of the donor’s RP4 plasmid, and that the antiepileptic drug carbamazepine significantly increased both the intragenera and intergenera conjugative transfer of the multiresistance genes.

### Carbamazepine induces over-production of ROS and stimulates an SOS response

**Fig. 2:**
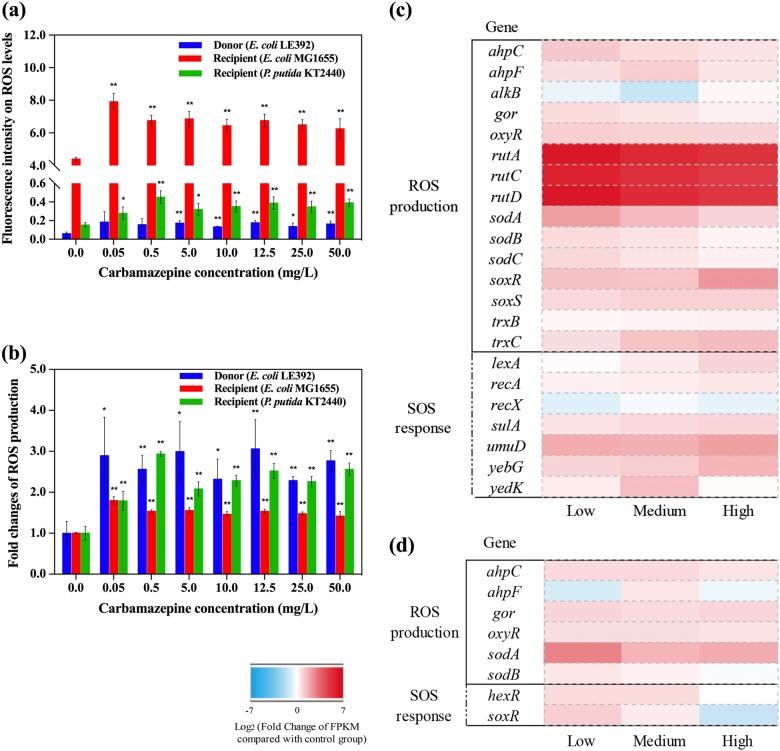
Effects of carbamazepine on ROS generation and SOS response in the donor (*E. coli* K-12 LE392) and recipient bacterial strains (*E. coli* MG1655 and *P. putida* KT2440). **a** Fluorescence intensity relating to ROS levels. **b** Fold changes of ROS production. **c** Fold changes of expression of core genes related to ROS production and SOS response in donor bacteria. **d** Fold changes of expression of core genes related to ROS production and SOS response in recipient bacteria. Significant differences between carbamazepine-dosed samples and the control were detected using independent-sample *t* test, *P* values were corrected by the “Benjamini–Hochberg” method as *P*_adj_, *P_adj_ < 0.05, and **P_adj_ < 0.01. Low, medium, and high in *X*-axis refer to 0.05, 10.0, and 50.0 mg/L carbamazepine, respectively

Other studies report that oxidative stress enhances the frequency of bacterial conjugative transfer [11, 31]. Consequently, we hypothesize that carbamazepine promotes conjugative transfer of ARGs by causing oxidative stress in bacteria. In this instance we measured the levels of ROS in the cells. These are highly reactive molecules, such as peroxides and hydroxyl radicals, that interfere with the normal functions of bacteria [32, 33]. It was seen that with the increased dosage of carbamazepine, ROS production in the donor and recipient strains increased (Fig. 2a). Low concentrations of carbamazepine were seen to significantly impact the ROS production. However, the increased levels of carbamazepine did not alter the ROS production much further (Fig. 2b and Fig. S6a). The relative fold changes of ROS production for the donor and the two recipients ranged from 1.4 to 3.1. To fully assess this effect, we tested still lower concentrations of carbamazepine on the production of ROS. It was seen that at carbamazepine levels equal to or lower than 0.0005 mg/L significant ROS generation was not induced (Fig. S6a).

We were able to reverse the effect of ROS on the conjugative frequency. With the addition of an ROS scavenger, thiourea, during the mating period, the conjugative transfer frequency decreased significantly (*P*_adj_ < 0.05) (Fig. 1b). For example, under the exposure of 50.0 mg/L carbamazepine, fold change in intergenera transfer decreased from 12-fold to less than 3-fold with the addition of thiourea. More obviously for the intragenera transfer, there was no any significant difference between the control and the addition of scavenger under carbamazepine exposure of 0.05–10 mg/L (*P*_adj_ > 0.05).

Changes in gene expression indicated both the donor and recipient bacterial strains responded quickly to carbamazepine. For the *E. coli* donor bacterial strain, the redox-sensing gene, *oxyR*, was upregulated significantly upon exposure to carbamazepine by 1.9- to 5.1-fold, in comparison to the control group (no carbamazepine exposure) (Fig. 2c) The gene *oxyR* is a regulator of genes involved in oxidative stress defence [34, 35]. It was seen that genes coding for superoxide dismutase (*sodA*), glutathione oxidoreductase (*gor*), and alkyl hydroperoxide reductase (*ahpC* and *ahpF*) had increased expression during exposure to carbamazepine. These are antioxidant enzymes and were likely overexpressed to protect the donor from the ROS attack [36]. We also detected changes of gene expression in the recipient bacterial strain *P. putida* during exposure to carbamazepine (Fig. 2d). Again the regulatory gene *oxyR* was seen to have increased expression by about 1.6-fold (Fig. 2d). Additionally, the expression of the genes *gor* and *ahpC* was also increased by about 1.7-fold greater than that in the control group.

When exposed to all levels of carbamazepine, ranging from environmental-relevant concentrations to 50.0 mg/L, genes relevant to the SOS response, *sulA*, *yedK*, *yebG*, and *umuD*, all showed increased expression. In particular the *umuD* gene in *E. coli* was upregulated more than 3.3-fold, and this has previously shown to play key roles in the SOS response to DNA damage [37]. It is seen that high levels of ROS can induce the SOS response in bacteria, which is a common global response to DNA damage [37]. Consequently, during this exposure to carbamazepine the levels of ROS increased and this likely caused DNA damage to the bacterial strains.

Thus, from these phenotypic and genotypic results, we find that carbamazepine increased the conjugative transfer of ARGs and this is partially due to increased oxidative stress and the stimulation of the SOS response in bacterial cells.

### Carbamazepine increases cell membrane permeability

**Fig. 3:**
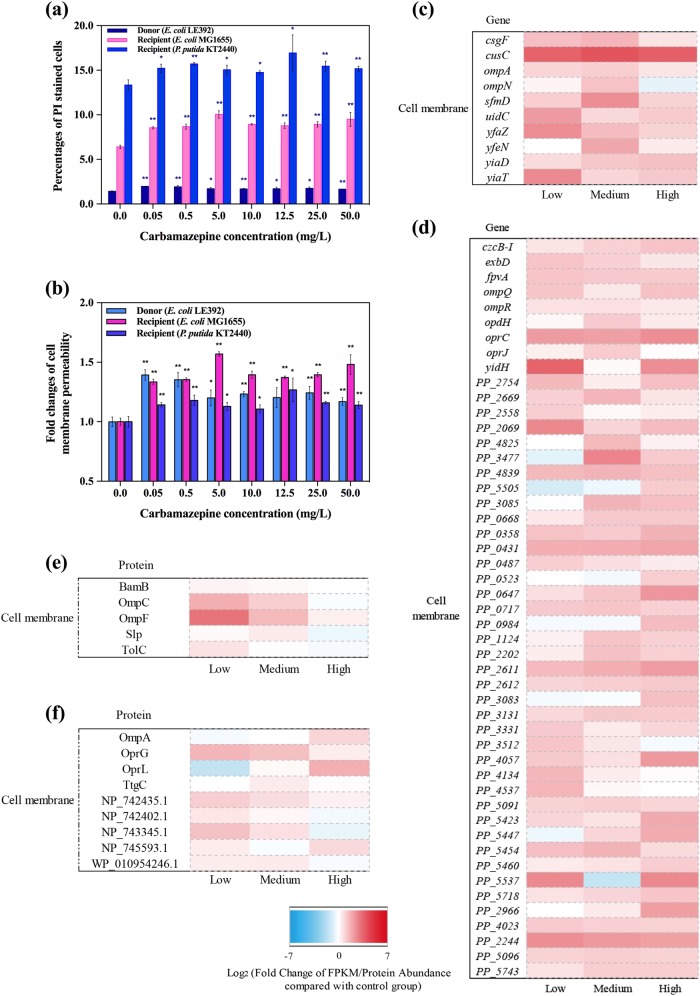
Effects of carbamazepine on cell membrane in donor (*E. coli* K-12 LE392) and recipient bacterial strains (*E. coli* MG1655 and *P. putida* KT2440). **a** Percentages of PI stained cells. **b** Fold changes of cell membrane permeability. **c** Fold changes of expression of core genes related to cell membrane in donor bacteria. **d** Fold changes of expression of core genes related to cell membrane in recipient bacteria. **e** Fold changes of abundance of core proteins related to cell membrane in donor bacteria. **f** Fold changes of abundance of core proteins related to cell membrane in recipient bacteria. Significant differences between carbamazepine-dosed samples and the control were detected using independent-sample *t* test, *P* values were corrected by the “Benjamini–Hochberg” method as *P*_adj_, *P_adj_ < 0.05, and **P_adj_ < 0.01. Low, medium, and high in X-axis refer to 0.05, 10.0, and 50.0 mg/L carbamazepine, respectively

Increased levels of ROS can also damage cell membranes, this can weaken these important barriers and render them more permeable to the transfer of genetic material [38]. External chemicals can enhance conjugative transfer frequency of genes by damaging cell membranes [11], or by increasing cell membrane permeability [12]. In this study, we hypothesize that damaged cell membranes play a key role in the carbamazepine-increased conjugation process. With exposure to carbamazepine (even as low as 0.05 mg/L) the cell membrane permeability of the donor and recipient bacterial strains increased significantly (*P*_adj_ < 0.05) (Fig. 3a). This permeability increased by 1.1- to 1.6- fold compared to that in the control cells (Fig. 3b). We further tested lower concentrations of carbamazepine for this effect, and it was seen that concentrations as low as 0.0005 mg/L could significantly increase the cell membrane permeability (Fig. S6b).

The increased membrane permeability induced by carbamazepine is also supported by the gene expression and protein abundance analyses we performed. Genes relevant to the outer cell membrane permeability were upregulated during the exposure to carbamazepine. For example, both major outer membrane protein regulator genes, *ompA* and *ompN*, were upregulated in the donor bacteria (Fig. 3c). The highest expression of these genes was at exposure to 10.0 mg/L carbamazepine, and this correlates with the high intergenera transfer frequency we detected. At higher levels of carbamazepine (50.0 mg/L) the expression of *ompN* decreased, and this may be due to possible toxic effects of the high concentration [39]. The corresponding membrane proteins also showed altered expression in the presence of carbamazepine. For example, 9.4- and 3.4-fold increases of OmpF and OmpC proteins were observed in the donor bacteria at 0.05 mg/L of carbamazepine. These proteins are important for regulating cell membrane permeability [40]. Genes responsible for cell membrane conditions and components in the recipient bacteria, *ompQ*, *opdH*, and *oprJ*, were upregulated as well. More than a 3-fold increase of the protein OprG was observed under the exposure of 0.05 mg/L carbamazepine (Fig. 3d). The OprG protein plays a key role in outer membrane channels and changes [41].

In addition, putative outer membrane related genes in the donor bacteria, such as *sfmD*, *yfeN*, *csgF*, *yfaZ*, *yiaD*, and *uidC*, also exhibited enhanced expression levels. Compared with the control group, under the exposure of 0.05, 10.0, and 50.0 mg/L carbamazepine, the expression levels of these genes increased, ranging from 1.7 to 6.1-, 1.8 to 5.9-, and 1.4 to 2.7-fold, respectively (Fig. 3c). In the donor bacteria it seems the transcription of these genes responded sharply to the low level exposure (0.05 mg/L) of carbamazepine. These genes are coding for outer membrane proteins, which means the increased expression would potentially play a role in increased cell membrane permeability [42]. This is consistent with the phenotypic results, where the low concentrations of carbamazepine significantly enhanced the conjugative transfer frequency. The gene *cusC*, was highly upregulated at all levels of carbamazepine, which was as high as a 16.9-fold increase. This is an outer membrane transport protein, a key element in the bacterial copper/silver efflux system [42, 43].

Similarly, in the recipient bacteria, many cell membrane related genes were also upregulated. This includes the genes *ompQ*, *opdH*, and *oprJ* (Fig. 3d). Additionally, outer membrane porins, including OprG, had increased abundance when exposed to carbamazepine (Fig. 3f). The increased expression of these elements would potentially contribute to an increased membrane permeability [42].

### Carbamazepine affects the expression of conjugation genes in the RP4 plasmid

**Fig. 4:**
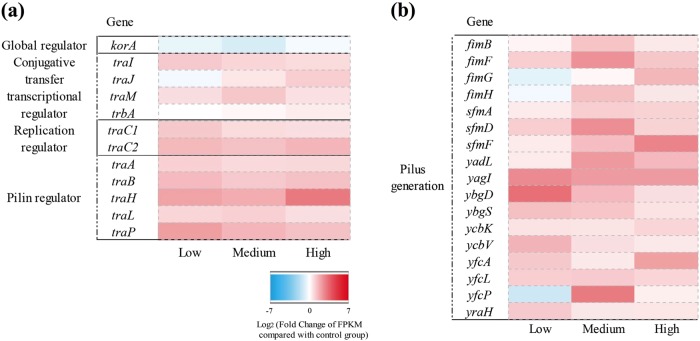
Effects of carbamazepine on pilus generation in donor bacteria (*E. coli* K-12 LE392) and conjugative plasmid (IncP α-type RP4 plasmid). **a** Fold changes of expression of core genes in RP4 plasmid. **b** Fold changes of expression of core genes related to adhesive-pilus generation in donor bacteria. Low, medium, and high refer to 0.05, 10.0, and 50.0 mg/L carbamazepine, respectively

During the conjugative transfer the plasmid undergoes a continuing process of replication, partitioning, and conjugation [44]. Genes on the RP4 plasmid that are important for the continual conjugation process include the major global regulatory gene, *korA*, the conjugative transfer transcriptional regulators *traI*, *traJ*, *traM*, and the replication related gene *traC* [45]. Under the exposure of carbamazepine, expression of the regulatory gene *korA* was repressed compared to that in the control group. As a result, the conjugative transfer transcriptional regulator genes and the replication genes showed increased expression during carbamazepine exposure (Fig. 4b). At the low level exposure of 0.05 mg/L carbamazepine, both replication primase genes, *traC1* and *traC2*, were upregulated more than 2.3-fold. Higher levels of carbamazepine did not further increase the expression levels of these genes. Thus, the low dosage of carbamazepine was seen to induce conjugation activity from the RP4 plasmid.

During conjugation the plasmid is transferred from donor to recipient bacteria through a pilin bridge [5]. Pili formation genes on the RP4 plasmid include *traB*, *traH*, *traP*, *traA*, and *traL*, and these form the mating pair formation (Mpf) system [46]. During this carbamazepine exposure, most genes for pilin formation in the RP4 plasmid were upregulated (Fig. 4a). For example, compared to the control group, the gene *traB* was upregulated 2.8-, 2.2-, 2.6- fold at the carbamazepine levels of 0.05, 10.0, 50.0 mg/L, respectively. As well the *traH* gene expression increased by up to 8.6-fold at the carbamazepine concentration of 50.0 mg/L. Considering these changed transcriptional levels of the RP4 plasmid genes, we propose that carbamazepine enhanced the conjugative transfer by inducing increased expression of the pilin production genes, and by altering expression of the conjugative regulators, which would accelerate the plasmid replication.

In addition, in the conjugative process, direct cell-to cell contact is necessary for the plasmid transfer [5]. In the donor bacterial strain, *E. coli* K-12, some *fim*-like operons, including the *yag* or *mat* operons, have been characterized to be involved in adhesion and colonization [47]. In our study, under the exposure of carbamazepine, genes in these adhesion-relevant operons were significantly upregulated. For example, compared with the control group, expression of the *yagI* gene was increased by as much as 6.1-fold with exposure to carbamazepine (Fig. 4b).

## Discussion

The spread of antibiotic resistance is a serious global threat for public health and the ecology of the environment. Previous studies demonstrate that sublethal levels of antibiotics can behave as selective drivers, and thus facilitate wide dissemination of ARGs [48–50]. Apart from antibiotics, non-antibiotic pharmaceuticals also form a great proportion of the total pharmaceutical consumption. The antiepileptic drug, carbamazepine, has high worldwide use and as it is resistant to biodegradation it is frequently detected at relatively high levels in our aquatic environments [16]. Here we show that a non-antibiotic pharmaceutical, carbamazepine, can significantly promote the dissemination of antibiotic resistance. This study demonstrates carbamazepine enhances horizontal transfer of multiresistance genes borne by the RP4 plasmid within and across bacterial genera. Very recently, Maier et al. [51] reported that over 200 human-targeted drugs might pose antibiotic-like side effects in gut bacterial strains by screening more than 1 000 drugs. Nevertheless, they did not find carbamazepine have any inhibitory or biocidal effects on the selected bacterial strains, under the exposure of 20 µM carbamazepine. Our study represents a critically important addition that is not covered in the research by Maier et al. [51].

Our results showed that under laboratory conditions the spontaneous conjugative transfer frequency of RP4 was low, after 8-h mating, at about 6.85 × 10^−5^ for intragenera transfer, and 2.67 × 10^−6^ for intergenera transfer. These are similar frequencies to that detected in other studies [11, 52]. However, in the presence of carbamazepine the conjugative transfer was enhanced significantly. During the donor-recipient mating with 50.0 mg/L of carbamazepine the conjugative frequencies increased 9 times for intragenera and 12 times for intergenera transfer. In comparison to other environmental contaminants or applied treatments, the conjugative transfer frequencies facilitated by carbamazepine were lower than that induced by 0.50 g/L nanoalumina [11] or by 0.5 g/L ionic liquid [BMIm][PF6] [14], but they were higher than conjugative frequencies induced by disinfectants [21]. Importantly, in this study, to observe the effect of carbamazepine we used levels that were at environmentally relevant concentrations [53]. Noticeably, at these low levels we still detected significantly increased transfer of the ARGs. The reverse transfer experiment, where *P. putida* KT2440 with RP4 plasmid was the donor and *E. coli* MG1655 was the recipient, also showed that carbamazepine at low concentrations significantly enhanced conjugative transfer of ARGs. There is evidence that frequencies of conjugative transfer in natural biofilms may be several orders magnitude higher than those under laboratory conditions with planktonic cells. This was detected during investigations where microplastic pollution increased gene exchange in natural aquatic ecosystems [54]. Therefore, the conjugative transfer frequency promoted by carbamazepine is likely to be higher in the real environment.

In addition to the increased transfer frequencies we detected, we further verified that it was the RP4 plasmid being transferred. Randomly selected transconjugants were subjected to antibiotic MIC tests, plasmid electrophoresis, as well as plasmid sequencing. These all supported our conclusion that carbamazepine enhanced the horizontal transfer of ARGs.

**Fig. 5:**
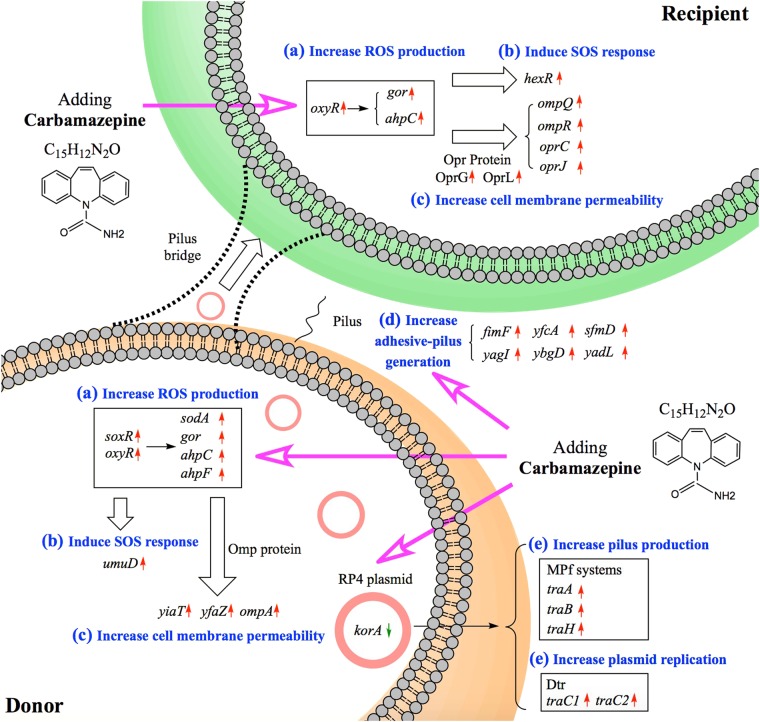
Possible mechanisms underlying the phenomenon that carbamazepine accelerates conjugative transfer of ARGs borne by RP4 plasmid. **a** Carbamazepine induces over-production of ROS in both donor and recipient bacteria. **b** Carbamazepine stimulates SOS response in both donor and recipient bacteria. **c** Carbamazepine increases cell membrane permeability in both donor and recipient bacteria. **d** Adhesive-pilus generation is enhanced by carbamazepine in donor bacterial strain. **e** Conjugative and replicative genes in RP4 plasmid are affected under the exposure of carbamazepine

We also investigated potential mechanisms to explain the role of carbamazepine in the enhancement of conjugative transfer of the RP4 plasmid. In contrast to other studies [11, 14, 21], we employed multiple investigative approaches to disclose the underlying mechanisms. This included flow cytometry to measure membrane permeability and oxidative stress, MinION plasmid DNA sequencing, gene expression analysis by whole-genome RNA sequencing, and quantitative proteomic response analysis. Based on the phenotypic and genotypic data, we summarized the mechanisms underlying the carbamazepine-enhanced conjugative transfer of the RP4 plasmid (Fig. 5). We suggest there are three factors playing key roles in the conjugation process. These are the generation of ROS, the increased permeability of cell membranes of both donor and recipient bacteria, and the increased conjugation activity of the RP4 plasmid [38, 55].

Increased level of ROS is a main reason for carbamazepine-enhanced conjugation. With increased dosage of carbamazepine, the ROS production increased significantly (*P*_adj_ < 0.05) in both donor and recipient cells. This may be attributed to carbamazepine inhibiting the activities of antioxidant enzymes [56]. It is seen that carbamazepine induces increased oxidative stress in humans or animals by inhibiting antioxidant enzymes, such as inhibiting activities of superoxide dismutase, catalase, glutathione peroxidase, and glutathione reductase [57, 58]. Thus, we suggest carbamazepine could induce this oxidative stress at the low concentrations tested in this study (down to 0.005 mg/L). Moreover, when the experiment included the addition of an ROS scavenger, thiourea, the frequencies decreased significantly (*P*_adj_ < 0.05) for both intergenera and intragenera transfer. Thus, further supporting that ROS induced by carbamazepine is a main factor influencing the increased conjugation. It is known that bacterial strains respond quickly to oxidative stress to protect against ROS attack [36]. Indeed, in the presence of carbamazepine we detected increased expression of the oxidative regulators, *oxyR* and *soxR*, which woud upregulate expression of antioxidant genes, such as *sodA*, *gor*, *ahpC* [34, 35]. We propose that changes in ROS production is a main mechanism caused by carbamazepine that significantly enhances the conjugal transfer of the RP4 plasmid.

We also detected that the SOS response was activated in the presence of carbamazepine. This was possibly due to DNA damage caused by increased levels of ROS, and this was evident from the increased expression of the genes *sulA*, *umuD*, and *hexR* [37]. The SOS response has previously been reported to promote horizontal dissemination of ARGs [31, 49, 59]. However, conversely, it is also reported that conjugative transfer of ssDNA can induce the bacterial SOS stress response [60]. Thus, it is not clear whether the SOS response is a cause or a symptom of conjugative transfer activity.

In our study we found under the exposure of carbamazepine that the cell membrane permeability also increased significantly based on the PI stain (*P*_adj_ < 0.05). Relevant genes were also upregulated based on both transcriptional and translational levels. This included the key genes *omp*, *yia*, *yfaZ*, and *opr* [42, 61]. Also, the proteins OmpC and OmpF, which control cell membrane permeability [42, 62], were significantly in higher abundance during carbamazepine exposure. Donor and recipient bacteria responded quickly to even the lowest dosage of carbamazepine in our study, and this may partially explain why carbamazepine can facilitate significant conjugative transfer of the plasmid-borne ARGs. Based on these findings, we imply that changes in the cell membrane integrity is another mechanism by which carbamazepine enhanced the conjugative transfer of these ARGs.

This study examined the changes of the conjugative transfer in Gram-negative bacteria. Due to the many fundamental differences in bacterial membranes and outer membrane components, such altered transfer frequencies we detected here is likely to differ in other bacteria, for example in the Gram-positive bacteria.

During the conjugation process, physical cell-to-cell contact is necessary, which enables plasmid DNA to be transferred from donor to recipient bacteria. In donor *E. coli* K-12, Type 1 pili are demonstrated to increase the adhesion to adjacent cells [63]. In our study, most adhesion-relevant genes (e.g., *fim* and *yag*) were upregulated on exposure to carbamazepine. Thus, this implicates increased adhesive-pilus generation which could facilitate cell contact between donor and recipients, and enhance the plasmid DNA transfer. This increased cell contact could contribute to the increased conjugal transfer induced by carbamazepine.

The RP4 plasmid is a broad-host-range conjugative IncP α-type plasmid, that uses the plasmid DNA transfer and replication system, called Dtr, together with Mpf systems for conjugation [46]. The Dtr system is responsible for plasmid replication, while the Mpf system is essential for production of pilus. Pilus can form a pilin bridge, via which plasmid DNA can be transferred from donor to recipient bacteria. Pilus may work as a needle, thrusting the substrate proteins across one or several membrane barriers into the recipient cytoplasm [64]. It is also proposed that the Mpf system can serve to establish a trans-envelope channel structure, which is like a conduit for plasmid transfer between donor and recipient bacteria [65, 66]. In this study we found that carbamazepine significantly increased expression levels of genes belonging to the Dtr (*traC*) and Mpf systems (*traA*, *traB*, *traH*). As high as 8.6-fold increase in this gene expression was observed during the carbamazepine exposure. The enhanced expression levels may occur due to the influence of the global regulator *korA*. It is known that *korA* can regulate the replication, transfer and stable inheritance of plasmid genes. Specifically, repression of *korA* activates the Mpf systems, and also increases expression levels of *trfA*, which is responsible for plasmid replication [67]. Therefore, regulating conjugative gene expression in RP4 plasmid is another important way that carbamazepine is affecting the dissemination of ARGs.

## Conclusion and outlook

In this study, we show that carbamazepine, as one of the most detected non-antibiotic pharmaceuticals contaminating the water environment, significantly promotes the conjugative transfer of ARGs. Increased levels of ROS, activation of the SOS response, enhanced cell membrane permeability, and generation of pilus are potentially all contributing here to the higher transfer efficiency caused by carbamazepine. This is the first study on the dissemination of ARGs through HGT promoted by a non-antibiotic pharmaceutical. The findings here are an early warning to re-think and re-evaluate the potential antibiotic-like roles induced by non-antibiotic pharmaceuticals in environmental settings. The conjugation process used in this study can be seen as a model gene transfer system for environmental ecosystems. Further studies could be applied to test whether non-antibiotic pharmaceuticals are able to promote conjugation in mixed culture-based systems, for example in activated sludge, soils, and human guts in vivo.

## Electronic supplementary material

Supporting information
