# Supplementary material for: Antiepileptic drug carbamazepine promotes horizontal transfer of plasmid-borne multi-antibiotic resistance genes within and across bacterial genera
Source: ISME J. 2018 Oct 5;13(2):509–22. doi: 10.1038/s41396-018-0275-x (PMC6331567; doi:10.1038/s41396-018-0275-x)
Supplement: Supplementary file 1 — Supporting information [file 41396_2018_275_MOESM1_ESM.pdf]

Supporting information

Antiepileptic drug carbamazepine promotes horizontal transfer of plasmid-borne multi-antibiotic resistance genes within and across bacterial genera

Running Title: Horizontal gene transfer enhanced by carbamazepine

Yue Wang, Ji Lu, Likai Mao, Jie Li, Zhiguo Yuan, Philip L. Bond, Jianhua Guo*

Advanced Water Management Centre, The University of Queensland, St. Lucia, Brisbane, Queensland, Australia, 4072

* Corresponding author: j.guo@awmc.uq.edu.au

This file includes:

Supplementary Texts 1 to 7

Supplementary Figures 1 to 7

Supplementary Tables 1 to 17

Supplementary References

Supplementary Methods

Text S1. Culture conditions for donor and recipient

Both donor and recipient were cultured separately in Luria-Bertani (LB) broth (pH 7.0) at 30 ^{o}C for 16 h with the supplementary of appropriate antibiotics. For donor, 17.0 mg/L tetracycline (Tet), 33.0 mg/L Kanamycin (Ka), and 100.0 mg/L ampicillin (Amp) were added, while 17.0 mg/L chloramphenicol (Chl) was dosed in the LB broth for recipient. After culturing, both donor and recipient were washed with phosphate-buffered saline (PBS, pH=7.2) twice to eliminate the possible influence induced by culture media. Afterwards, the donor and recipient were re-suspended separately in different volumes of PBS to obtain initial concentration of 10^8 cfu/ mL based on OD600 values (the relationships between OD600 and donor and recipient's concentration were predetermined, the results not shown). Then, the donor and recipient were mixed with the ratio of 1:1. The mixtures were applied immediately for the conjugation experiment.

Text S2. Selection plates for transconjugant and recipient

The selection plates for transconjugant contained all of the four kinds of antibiotics, while those for recipient only contained Chl. Concentrations of the antibiotics were the same as those in the culture media of donor and recipient. i.e., selection plates for transconjugant: 17.0 mg/L Tet, 33.0 mg/L Ka, 100.0 mg/L Amp, and 17.0 mg/L Chl, and selection plates for recipient contained 17.0 mg/L Chl. The results of selection plates are shown as cfu/mL and transfer frequency. All the selection plates were performed at least in triplicate.

Text S3. Bacterial growth curves of donor, recipients and transconjugants

Bacterial growth curves of donor, recipients and randomly-selected transconjugants were performed under the exposure of different concentrations of carbamazepine. OD_{600} was monitored hourly. Growth curve fitting and parameter calculation (including both the lag

time and maximum growth rate) were determined according to modified Gompertz Model as described previously [1]. Biological triplicate experiments were conducted under each condition.

Text S4. Detection of relative oxidative stress (ROS) and cell membrane permeability

Bacteria strains were washed twice with PBS and resuspended in PBS to 10^6 cfu/mL. For ROS detection, bacteria strains were incubated in dark at 37 ^{o}C for 30 min with 2', 7'-dichlorofluorescein diacetate (DCFDA, at a final concentration of 20 uM, abcam®). Then, 100 μL of the bacteria stained with DCFDA were treated with different concentrations of carbamazepine. 1.5% H_2O_2 was set as positive control, and ethanol was set as negative control. After complete mixing, the mixtures were incubated in dark at 25 ^{o}C for 2 h before measurement at 488 nm. As for cell membrane permeability detection, 100 μL of bacteria strain was exposed to different concentrations of carbamazepine, and incubated at 25 ^{o}C for 2 h. The same volume of ethanol was the negative control, while bacteria strain treated with 100 ^{o}C water was the positive control. The strains were then stained with 1 μL of propidium iodide (PI, 2 mM, Life Technologies) and incubated in the dark for 15 min before measurement at 561 nm. All data was analysed with CytExpert. Data were presented in a dot plot, in which upper right quadrant indicated DCFDA or PI positive cells (with increased ROS or cell membrane permeability), and upper left quadrant was normal cells. All the detections were conducted in triplicate. Relative fold increases in ROS production or cell membrane permeability were calculated as carbamazepine treated samples divided by negative control samples according to previous studies [2].

Text S5. MinION nanopore sequencing and analysis

Sequencing library for RP4 plasmid was established following the protocol 1D Native barcoding genomic DNA, EXP-NBD103 and SQK-LSK108. Briefly, core steps include end-

repair, purification (with AMPure XP beads), ligation of barcodes (1 different barcode per plasmid) using a ligase Master mix, purification with AMPure XP beads, pool barcoded plasmids, ligation of sequencing adapter, purification with AMPure XP beads and elution with ELB. The coordinates of the alignments, as well as identity and scores were analysed based on BLAST n according to previous research [3].

Text S6. RNA data analysis

Bioinformatics for whole-genome RNA sequencing were according to previous research [4]. Basically, NGS QC Toolkit (v2.3.3), SeqAlto (version 0.5), and Cufflinks (version 2.2.1) were applied to treat the raw sequence reads and to analyse the differential expression for triplicated samples. CummeRbund package in R was used to conduct the statistical analyses and visualization. Gene expression was calculated as fragments per kilobase of a gene per million mapped reads (FPKM). Differences in fold changes between different groups were calculated by log_2 fold-change (LFC) between control and carbamazepine-treated samples.

Text S7. Proteomics analysis

IDA data were combined and searched using ProteinPilot software, with the combined databases of *E. coli* SP only (received from Uniprot on 15th of October 2017) and *P. putida* KT2440 (received from NCBI on 15th of October 2017), Search setting for enzyme digestion was set to Trypsin and alkylation was set to iodoacetamide. Afterwards, the constructed IDA library and SWATH-MS data were loaded into PeakView v2.1 for further processing, with the peptide confidence threshold of 99%, number of peptides per protein of 5, and number of transitions per peptide of 3. A minimum of 2 peptides and 3 transitions was used for quantitative analysis. A stringency cut-off of q value less than 0.01 was used to identify the proteins with significant different expression levels.

Supplementary Figures

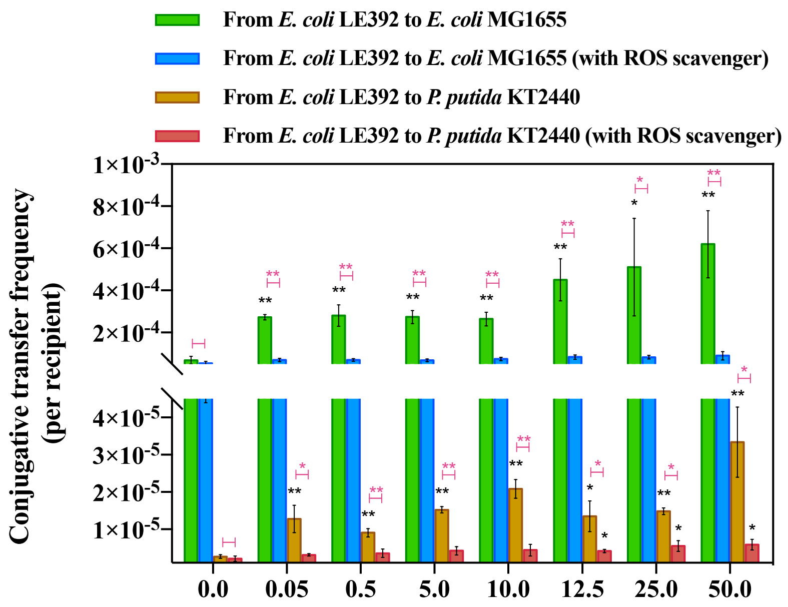

Fig. S1. Conjugative transfer frequency of ARGs induced by different concentrations of carbamazepine. Significant differences between carbamazepine-dosed samples and the control were tested using independent-sample *t* test, *P* values were corrected by “Benjamini-Hochberg” method as P_{adj}, * $P_{adj} < 0.05$ and ** $P_{adj} < 0.01$.

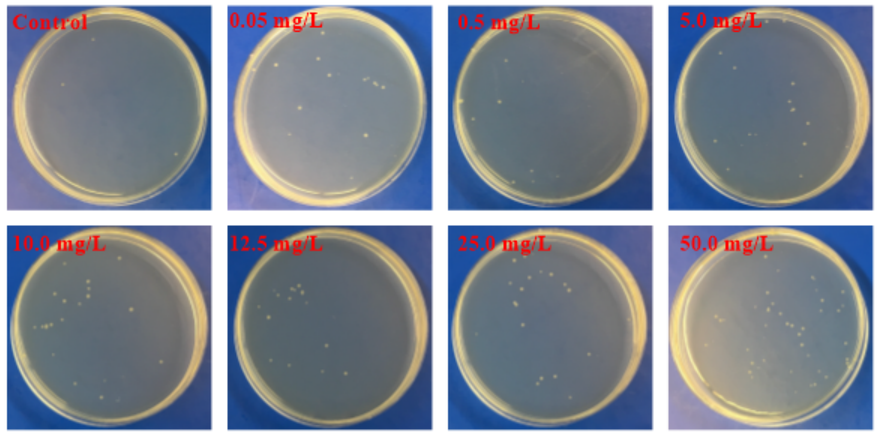

Fig. S2. Colonies growing on transconjugant-selective plates in intergenera transfer process

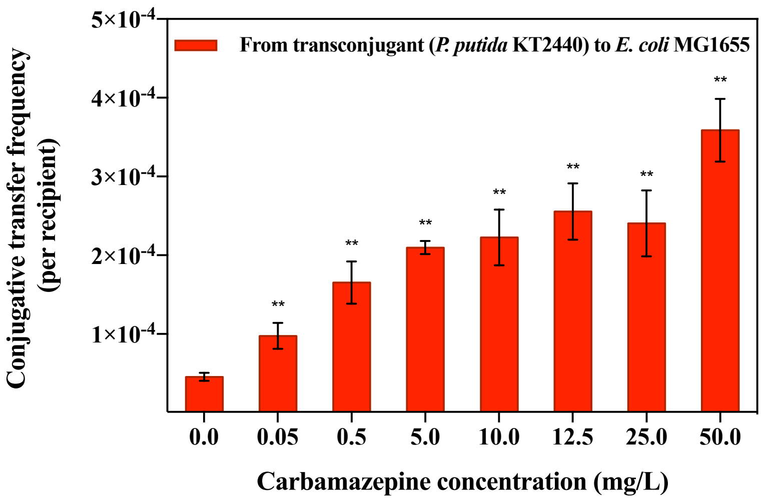

Fig. S3. Conjugative reverse transfer frequency of ARGs induced by different concentrations of carbamazepine. Significant differences between carbamazepine-dosed samples and the control were tested using independent-sample *t* test, *P* values were corrected by "Benjamini-Hochberg" method as P_{adj}, * $P_{adj} < 0.05$ and ** $P_{adj} < 0.01$.

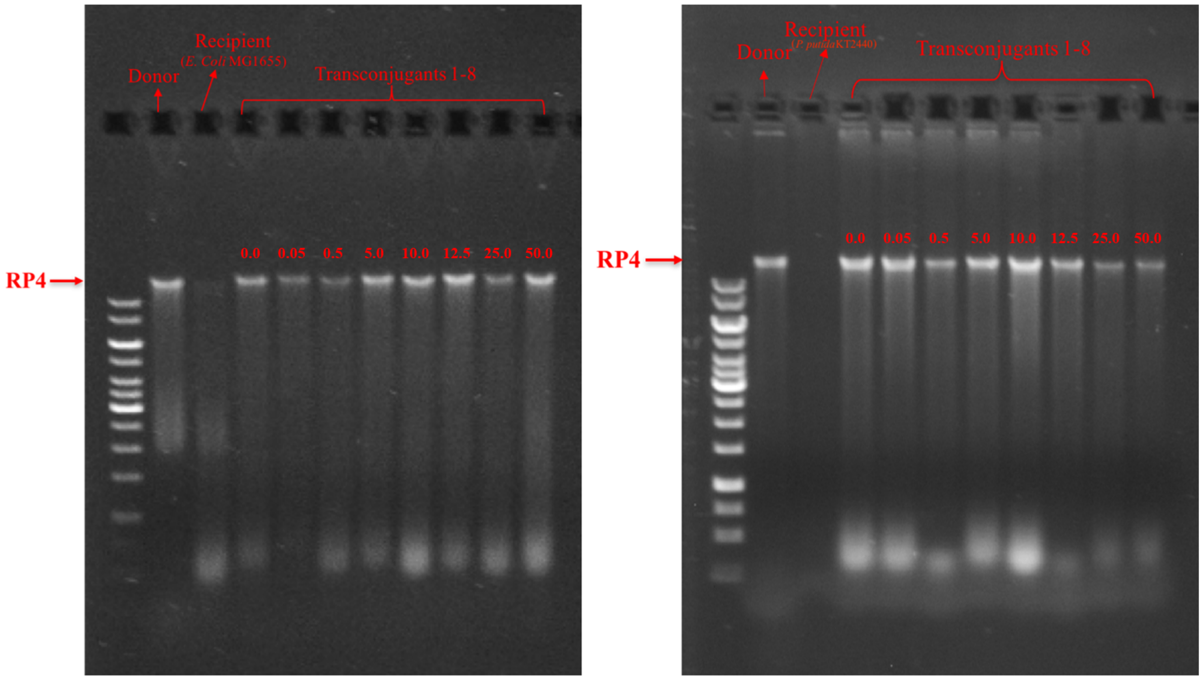

Fig. S4. Electrophoresis of plasmid extraction (Transconjugants 1-8: mating system treated with 0.0, 0.05, 0.5, 5.0, 10.0, 12.5, 25.0, 50.0 mg/L carbamazepine, respectively)

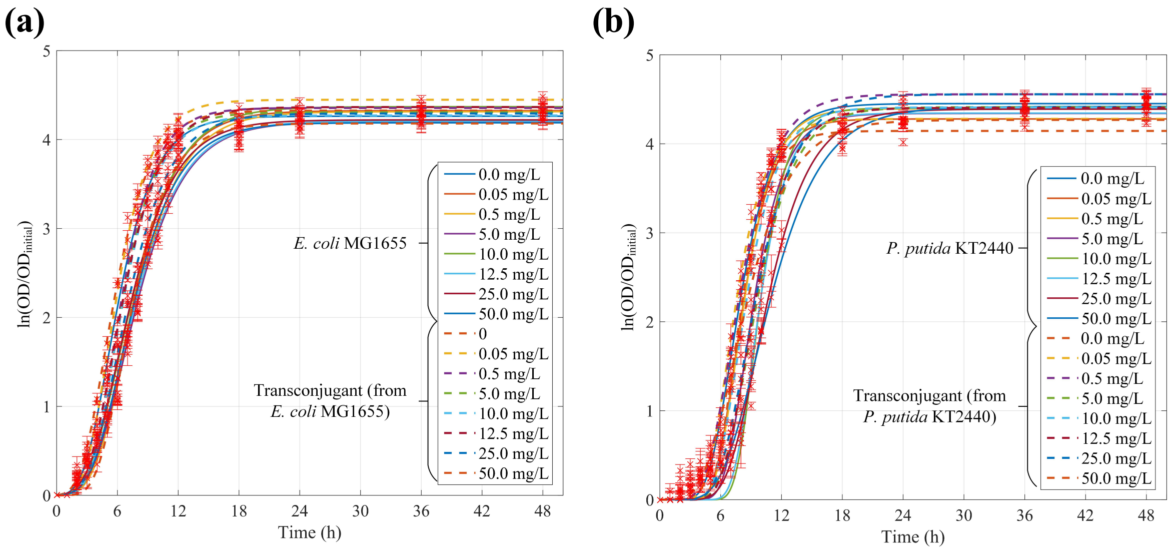

Fig. S5. Growth curves of recipients and their corresponding transconjugants during exposure to different levels of carbamazepine. (a) Recipient for intragenera transfer and the transconjugant (b) Recipient for intergenera transfer and the transconjugant.

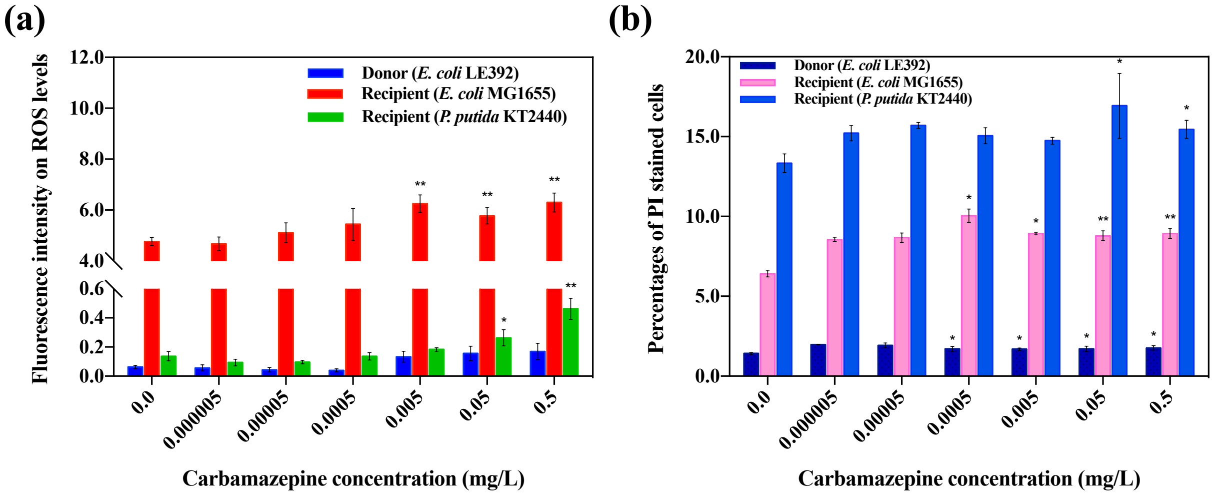

Fig. S6. Effects of carbamazepine on ROS generation and cell membrane in the donor (*E. coli* K-12 LE392) and recipient bacterial strains (*E. coli* MG1655 and *P. putida* KT2440). (a) Fluorescence intensity relating to ROS levels. (b) Percentages of PI stained cells. Significant differences between carbamazepine-dosed samples and the control were tested using independent-sample t test, P values were corrected by "Benjamini-Hochberg" method as P_{adj}, * $P_{adj} < 0.05$ and ** $P_{adj} < 0.01$.

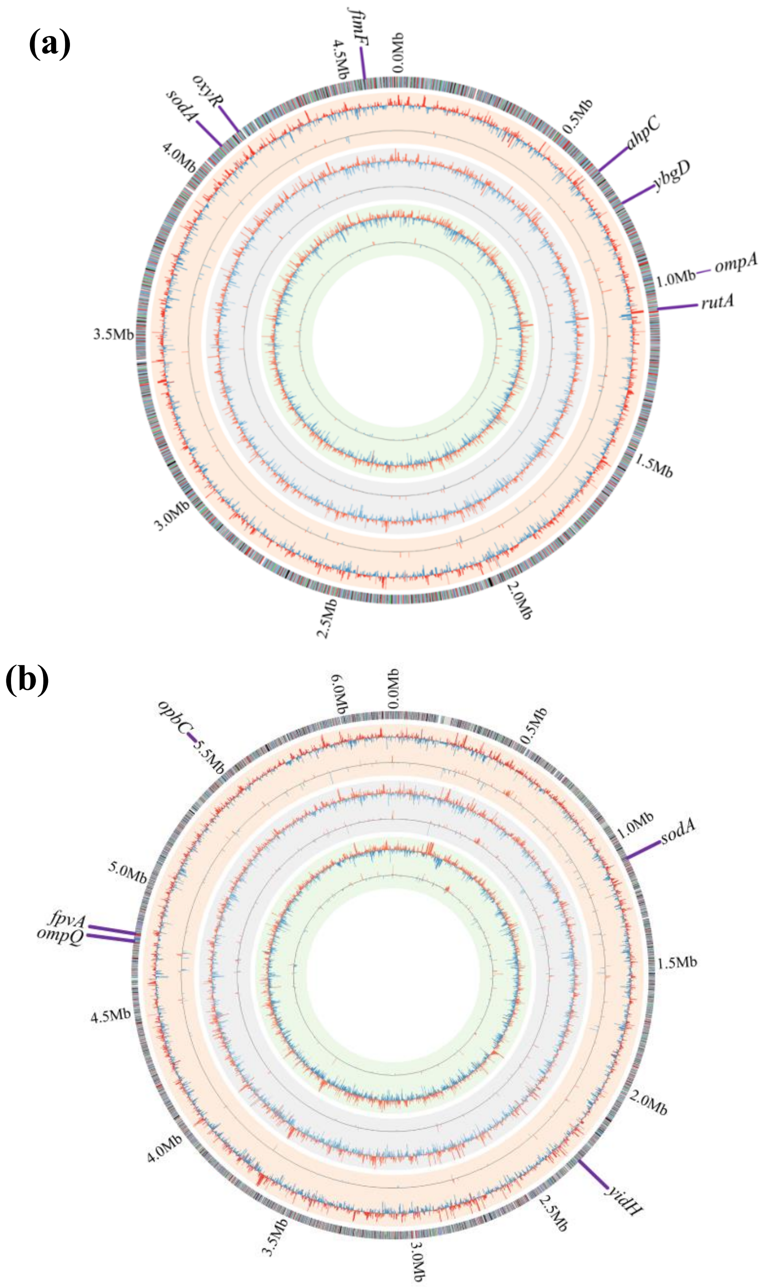

Fig. S7. The global transcriptome and proteome response of donor and recipient bacterial strain to carbamazepine exposure. (a) Donor bacterial strain *E. coli* K-12 LE392. (b) Recipient bacterial strain (*P. putida* KT2440). The outermost rings are reference genome of donor and recipient bacterial strains, respectively. The second and third rings (with orange background) are transcriptome and proteome response towards 0.05 mg/L carbamazepine. The fourth and fifth rings are transcriptome and proteome response towards 10.0 mg/L carbamazepine. The sixth and seventh rings are transcriptome and proteome response towards 50.0 mg/L carbamazepine. Core genes related to carbamazepine-accelerated conjugative transfer are highlighted along the genome.

Supplementary Tables

Table S1. Minimum inhibitory concentrations (MICs) of different transconjugants in intragenera transfer*

Antibiotics	MICs (μg/mL)									
	Donor	Recipient	TC 1	TC 2	TC 3	TC 4	TC 5	TC 6	TC 7	TC 8
Tet	10.24	6.82	15.36	15.36	15.36	15.36	15.36	15.36	15.36	15.36
Ka	20	2	30	30	30	30	30	30	30	30
Amp	>120	45	>120	>120	>120	>120	>120	>120	>120	>120
Chl	0.13	10.24	10.24	10.24	10.24	10.24	10.24	10.24	10.24	10.24

*TC 1-8: transconjugants in mating system treated with 0.0, 0.05, 0.5, 5.0, 10.0, 12.5, 25.0, 50.0 mg/L Carbamazepine, respectively

Table S2. Minimum inhibitory concentrations (MICs) of different transconjugants in intergenera transfer*

Antibiotics	MICs (μg/mL)									
	Donor	Recipient	TC 1	TC 2	TC 3	TC 4	TC 5	TC 6	TC 7	TC 8
Tet	10.24	1.28	10.24	10.24	10.24	10.24	10.24	10.24	10.24	10.24
Ka	20	5	20	20	20	20	20	20	20	20
Amp	>120	6.25	>120	>120	>120	>120	>120	>120	>120	>120
Chl	0.13	0.39	0.39	0.39	0.39	0.39	0.39	0.39	0.39	0.39

* TC 1-8: transconjugants in mating system treated with 0.0, 0.05, 0.5, 5.0, 10.0, 12.5, 25.0, 50.0 mg/L Carbamazepine, respectively

Table S3. Modelling results for growth curves of donor strain (*E. coli* LE392) under the exposure of carbamazepine[#]

Parameters	Carbamazepine Concentration (mg/L)							
	0	0.05	0.5	5	10	12.5	25	50
R^2	0.997±0.001	0.995±0.002	0.998±0.000	0.994±0.001	0.996±0.000	0.997±0.001	0.991±0.004	0.991±0.002
Maximum growth rate (h^{-1})	0.664±0.018	0.769±0.015**	0.696±0.019	0.724±0.011*	0.737±0.016*	0.730±0.040	0.652±0.015	0.660±0.017
Lag time (h)	2.722±0.207	2.925±0.054	3.124±0.181	3.081±0.194	3.772±0.152*	3.836±0.294*	3.403±0.125*	3.714±0.211*

[#] The modelling was based on modified Gompertz Model, Curve Fitting Tool in Matlab R2015b was applied. Significant differences were calculated between carbamazepine-dosed groups with the LB group using independent-sample *t* test, *P* values were corrected by "Benjamini-Hochberg" method as P_{adj}. ns: not significant, *: P_{adj}<0.05 and **: P_{adj}<0.01.

Table S4. Modelling results for growth curves of recipient strain (*E. coli* MG1655) under the exposure of carbamazepine*

Parameters	Carbamazepine Concentration (mg/L)							
	0	0.05	0.5	5	10	12.5	25	50
R^2	0.996±0.001	0.990±0.002	0.991±0.001	0.994±0.001	0.991±0.003	0.993±0.001	0.990±0.000	0.988±0.001
Maximum growth rate (h^{-1})	0.627±0.005	0.537±0.009**	0.509±0.007**	0.473±0.010**	0.509±0.007**	0.471±0.006**	0.507±0.027**	0.505±0.008**

Lag time (h)	2.613±0.032	3.527±0.207**	3.590±0.213**	3.355±0.268*	3.103±0.206*	3.191±0.178*	3.011±0.421	3.340±0.275*

[#] The modelling was based on modified Gompertz Model, Curve Fitting Tool in Matlab R2015b was applied. Significant differences were calculated between carbamazepine-dosed groups with the LB group using independent-sample *t* test, *P* values were corrected by "Benjamini-Hochberg" method as P_{adj}. ns: not significant, *: P_{adj}<0.05 and **: P_{adj}<0.01.

Table S5. Modelling results for growth curves of recipient strain (*P. putida* KT2440) under the exposure of carbamazepine*

Parameters	Carbamazepine Concentration (mg/L)							
	0	0.05	0.5	5	10	12.5	25	50
R^2	0.994±0.002	0.995±0.001	0.993±0.002	0.991±0.002	0.990±0.003	0.986±0.002	0.986±0.008	0.995±0.001
Maximum growth rate (h^{-1})	0.778±0.039	0.789±0.025*	0.807±0.036*	0.761±0.034	0.860±0.010**	0.840±0.072	0.507±0.101*	0.465±0.040**
Lag time (h)	4.745±0.168	5.428±0.131**	5.543±0.184**	6.331±0.162**	7.459±0.392**	7.227±0.239**	6.260±1.186	6.028±0.342*

[#] The modelling was based on modified Gompertz Model, Curve Fitting Tool in Matlab R2015b was applied. Significant differences were calculated between carbamazepine-dosed groups with the LB group using independent-sample *t* test, *P* values were corrected by "Benjamini-Hochberg" method as P_{adj}. ns: not significant, *: P_{adj}<0.05 and **: P_{adj}<0.01.

Table S6. Modelling results for growth curves of transconjugant strain from *E. coli* MG1655 under the exposure of carbamazepine*

Parameters	Carbamazepine Concentration (mg/L)							
	0	0.05	0.5	5	10	12.5	25	50
R^2	0.998±0.001	0.995±0.001	0.996±0.001	0.995±0.001	0.994±0.002	0.994±0.001	0.989±0.004	0.992±0.002
Maximum growth rate (h^{-1})	0.663±0.016	0.707±0.092	0.710±0.085	0.526±0.024**	0.531±0.012**	0.532±0.019**	0.506±0.006**	0.513±0.032**
Lag time (h)	2.341±0.208	2.781±0.046*	3.230±0.145**	3.218±0.210*	3.192±0.234*	3.004±0.169*	3.146±0.115**	3.911±0.290**
Significant difference compared with the recipient^	Maximum growth rate	ns	ns	ns	ns	ns	ns	ns
	Lag time	*	ns	ns	ns	ns	ns	ns

The modelling was based on modified Gompertz Model, Curve Fitting Tool in Matlab R2015b was applied. Significant differences were calculated between carbamazepine-dosed groups with the LB group using independent-sample *t* test, *P* values were corrected by "Benjamini-Hochberg" method as P_{adj}. ns: not significant, *: P_{adj}<0.05 and **: P_{adj}<0.01. ^ Compared with the maximum growth rate and lag time of the same concentration in Table S2.

Table S7. Modelling results for growth curves of transconjugant strain from *P. putida* KT2440 under the exposure of carbamazepine*

Parameters	Carbamazepine Concentration (mg/L)							
	0	0.05	0.5	5	10	12.5	25	50

R^2	0.993±0.003	0.995±0.001	0.994±0.002	0.991±0.002	0.990±0.001	0.993±0.001	0.990±0.001	0.991±0.002
Maximum growth rate (h^{-1})	0.799±0.013	0.782±0.016	0.778±0.027	0.732±0.025*	0.712±0.042	0.695±0.032*	0.660±0.019*	0.594±0.052**
Lag time (h)	4.408±0.245	4.633±0.108	5.073±0.343	5.381±1.180	6.099±0.770*	6.630±0.613*	6.147±0.942	6.140±0.337*
Significant difference compared with the recipient^	Maximum growth rate	ns	ns	ns	*	ns	ns	ns
	Lag time	**	ns	ns	ns	ns	ns	ns

The modelling was based on modified Gompertz Model, Curve Fitting Tool in Matlab R2015b was applied. Significant differences were calculated between carbamazepine-dosed groups with the LB group using independent-sample *t* test, *P* values were corrected by “Benjamini-Hochberg” method as P_{adj}. ns: not significant, *: P_{adj}<0.05 and **: P_{adj}<0.01. ^ Compared with the maximum growth rate and lag time of the same concentration in Table S3.

Table S8. Genes relevant to ROS production and SOS response in donor bacteria *E. coli* K-12 LE392 after exposure of carbamazepine

Gene	COG Annotation	Log_2(Fold Change of FPKM)[*]		
		Low-dosage	Medium-dosage	High-dosage
ahpC	Alkyl hydroperoxide reductase subunit AhpC (peroxiredoxin)	1.17	0.78	0.65
ahpF	Alkyl hydroperoxide reductase subunit AhpF	0.71	1.03	0.60
alkB	Alkylated DNA repair dioxygenase AlkB	-0.48[#]	-1.32[#]	0.19[#]
gor	Pyruvate/2-oxoglutarate dehydrogenase complex, dihydrolipoamide dehydrogenase (E3) component or related enzyme	0.75	0.58[#]	0.29[#]
oxyR	DNA-binding transcriptional regulator, LysR family	1.06	0.95	0.93
rutA	Flavin-dependent oxidoreductase, luciferase family (includes alkanesulfonate monooxygenase SsuD and methylene tetrahydromethanopterin reductase)	6.21	5.57	5.20
rutC	Enamine deaminase RidA, house cleaning of reactive enamine intermediates, YjgF/YER057c/UK114 family	5.87[#]	5.27[#]	4.87[#]

Gene	COG Annotation	Log2(Fold Change of FPKM)*		
		Low-dosage	Medium-dosage	High-dosage
rutD	Homoserine acetyltransferase	6.21#	5.45#	5.09#
sodA	Superoxide dismutase	2.15	1.50	0.92
sodB	Superoxide dismutase	0.75	0.63	0.25#
sodC	Cu/Zn superoxide dismutase	0.85	0.60#	0.30#
soxR	DNA-binding transcriptional regulator, MerR family	1.34#	1.27#	2.34#
soxS	AraC-type DNA-binding domain and AraC-containing proteins	0.81	1.01	0.98
trxB	Thioredoxin reductase	0.19#	0.25#	0.30#
trxC	Negative regulator of GroEL, contains thioredoxin-like and TPR-like domains	0.74#	1.30#	1.47#
lexA	SOS-response transcriptional repressor LexA (RecA-mediated autopeptidase)	0.10#	0.40#	0.91
recA	RecA/RadA recombinase	0.28#	0.34#	0.52

Gene	COG Annotation	Log$_2$(Fold Change of FPKM)[*]		
		**Low-dosage**	**Medium-dosage**	**High-dosage**
recX	SOS response regulatory protein OraA/RecX, interacts with RecA	-0.77[#]	-0.22[#]	-0.56[#]
sulA	Cell division inhibitor SulA, prevents FtsZ ring assembly	0.64	0.81	0.90
umuD	SOS-response transcriptional repressor LexA (RecA-mediated autopeptidase)	1.86[#]	1.72[#]	2.11[#]
yebG	dsDNA-binding SOS-regulon protein, induction by DNA damage requires cAMP	0.92	1.03	1.62
yedK	Putative SOS response-associated peptidase YedK	0.38[#]	1.45[#]	0.11[#]

[*]: Comparing with the control group without carbamazepine dosage

[#]: false discovery rate (FDR) > 0.05

Table S9. Proteins relevant to ROS production and SOS response in donor bacteria *E. coli* K-12 LE392 after exposure of carbamazepine

Protein	Gene description	Log_2(Fold Change of Protein Abundance)[*]		
		Low-dosage	Medium-dosage	High-dosage
AhpF	alkyl hydroperoxide reductase subunit F	-0.06	-0.20	-0.55
Gor	glutathione reductase	-0.74	-0.29	-0.23

[*]: Comparing with the control group without carbamazepine dosage

Table S10. Genes relevant to ROS production and SOS response in recipient bacteria *P. putida* KT2440 after exposure of carbamazepine

Gene	Gene description	Log_2(Fold Change of FPKM)[*]		
		Low-dosage	Medium-dosage	High-dosage
ahpC	peroxiredoxin/alkylhydroperoxide reductase small subunit	0.80	0.81	0.59[#]
ahpF	alkyl hydroperoxide reductase subunit F	-0.94[#]	0.57[#]	-0.40[#]
gor	glutathione reductase	0.93	0.79	0.88
oxyR	oxidative and nitrosative stress transcriptional dual regulator	0.74	0.72	0.61
sodA	superoxide dismutase	2.83[#]	1.63[#]	1.82[#]

Gene	Gene description	Log$_2$(Fold Change of FPKM)*		
		Low-dosage	Medium-dosage	High-dosage
sodB	superoxide dismutase	0.56	0.28#	-0.17#
hexR	DNA-binding transcriptional regulator	0.77#	0.77#	0.03#
soxR	DNA-binding transcriptional regulator	1.04#	0.41#	-1.34#

*: Comparing with the control group without carbamazepine dosage

#: false discovery rate (FDR) > 0.05

Table S11. Proteins relevant to ROS production and SOS response in recipient bacteria *P. putida* KT2440 after exposure of carbamazepine

Protein	Gene description	Log$_2$(Fold Change of Protein Abundance)*		
		Low-dosage	Medium-dosage	High-dosage
SodB	superoxide dismutase	-0.27	0.09	-0.85
NP_744587.1	peroxiredoxin/alkylhydroperoxide reductase small subunit	0.71	0.80	0.25

*: Comparing with the control group without carbamazepine dosage

Table S12. Genes relevant to cell membrane in donor bacteria *E. coli* K-12 LE392 after exposure of carbamazepine

**Gene**	**COG Annotation**	**Log_2(Fold Change of FPKM)[*]**		
		**Low-dosage**	**Medium-dosage**	**High-dosage**
csgF	----	1.38[#]	1.60[#]	0.60[#]
cusC	Outer membrane protein TolC	3.68[#]	4.08[#]	3.75[#]
ompA	Cell wall/membrane/envelope biogenesis	0.88	1.04	0.60
ompN	Cell wall/membrane/envelope biogenesis	0.24[#]	1.32[#]	-inf
sfmD	Cell motility; [W] Extracellular structures	1.07[#]	2.56[#]	0.95[#]
uidC	----	2.22[#]	0.83[#]	1.08[#]
yfaZ	----	2.58[#]	1.48[#]	0.96[#]
yfeN	----	-0.04[#]	1.94[#]	0.46[#]
yiaD	Outer membrane protein OmpA and related peptidoglycan-associated (lipo)proteins	0.77[#]	1.20[#]	1.41[#]
yiaT	Cell wall/membrane/envelope biogenesis	2.61[#]	0.87[#]	1.16[#]

[*]: Comparing with the control group without carbamazepine dosage

[#]: false discovery rate (FDR) > 0.05

Table S13. Proteins relevant to cell membrane in donor bacteria *E. coli* K-12 LE392 after exposure of carbamazepine

**Protein**	**Protein description**	**Log_2(Fold Change of Protein Abundance)***		
		**Low-dosage**	**Medium-dosage**	**High-dosage**
BamB	Outer membrane protein assembly factor BamB	0.25	0.14	-0.03
OmpC	Outer membrane protein C (Outer membrane protein 1B) (Porin OmpC)	1.77	1.03	-0.15
OmpF	Outer membrane protein F (Outer membrane protein 1A) (Outer membrane protein B) (Outer membrane protein IA) (Porin OmpF)	3.23	1.51	0.32
Slp	Outer membrane protein Slp	0.13	0.46	-0.41
TolC	Outer membrane protein TolC (Multidrug efflux pump subunit TolC) (Outer membrane factor TolC)	0.61	-0.08	-0.22

*: Comparing with the control group without carbamazepine dosage

Table S14. Genes relevant to cell membrane in recipient bacteria *P. putida* KT2440 after exposure of carbamazepine

Gene	Gene description	Log$_2$(Fold Change of FPKM)[*]		
		**Low-dosage**	**Medium-dosage**	**High-dosage**
czcB-I	Function of homologous gene experimentally demonstrated in an other organism%3B Product type m: membrane component%3B Transport and binding proteins	0.60[#]	0.98[#]	1.28
exbD	TonB-gated outer membrane transporter gating inner membrane protein	1.27	0.97[#]	0.54[#]
fpvA	TonB-dependent outer membrane ferripyoverdine receptor FpvA	1.25	1.15	1.09
ompQ	outer membrane pyoverdine efflux protein	1.25[#]	0.52[#]	1.32[#]
ompR	two-component system DNA-binding response regulator	0.64	0.66	0.48[#]
opdH	tricarboxylate-specific outer membrane porin	0.17[#]	1.10[#]	0.45[#]
oprC	copper receptor OprC	2.20	2.12	2.50
oprJ	outer membrane protein OprJ	0.31[#]	1.08[#]	0.07[#]
yidH	Putative membrane component	3.63[#]	0.12[#]	2.56[#]

Gene	Gene description	Log_2(Fold Change of FPKM)*		
		Low-dosage	Medium-dosage	High-dosage
PP_2754	OprD family outer membrane porin	1.51#	0.37#	1.40#
PP_2669	outer membrane protein	0.98#	1.64#	0.59#
PP_2558	outer membrane efflux protein	1.06#	0.15#	0.33#
PP_2069	multidrug MFS transporter outer membrane protein	2.64#	0.93#	1.48#
PP_4825	MarC family membrane protein	0.03#	1.52#	0.28#
PP_3477	type II secretion protein	-0.63#	2.80#	1.11#
PP_4839	iron-regulated membrane protein	1.53	1.66	1.42
PP_5505	Transmembrane protein	-inf	-0.40#	1.08#
PP_3085	transmembrane sensor	-0.10#	1.65#	1.38#
PP_0668	transmembrane sensor	0.48#	1.13#	1.14#
PP_0358	Membrane protein	1.32#	1.11#	1.68
PP_0431	Membrane protein	1.78	1.81	1.99
PP_0487	Membrane protein	1.07#	0.69#	0.39#
PP_0523	Membrane protein	-0.07#	-0.30#	1.03#

Gene	Gene description	Log_2(Fold Change of FPKM)*		
		Low-dosage	Medium-dosage	High-dosage
PP_0647	Membrane protein	0.70#	1.25#	2.35#
PP_0717	Membrane protein	1.13#	1.26#	0.98#
PP_0984	Membrane protein	-0.22#	-0.24#	1.48#
PP_1124	Membrane protein	0.29#	1.31#	1.01#
PP_2202	Membrane protein	0.37#	1.35#	0.99#
PP_2611	Membrane protein	1.50#	1.77#	2.18#
PP_2612	Membrane protein	0.91#	1.03#	1.18#
PP_3083	Membrane protein	-0.27#	-0.15#	1.41#
PP_3131	Membrane protein	0.85#	1.16#	1.08#
PP_3331	Membrane protein	1.14#	0.42#	0.89#
PP_3512	Membrane protein	1.25#	0.54#	-0.27#
PP_4057	Membrane protein	1.16#	0.67#	2.27#
PP_4134	Membrane protein	1.58#	0.38#	0.09#
PP_4537	Membrane protein	1.62#	0.17#	-0.008#

Gene	Gene description	Log_2(Fold Change of FPKM)[*]		
		Low-dosage	Medium-dosage	High-dosage
PP_5091	Membrane protein	0.96	1.02	0.88
PP_5423	Membrane protein	0.87[#]	0.63[#]	1.89[#]
PP_5447	Membrane protein	-0.34[#]	0.87[#]	1.82[#]
PP_5454	Membrane protein	1.40[#]	1.70[#]	0.79[#]
PP_5460	Membrane protein	0.42[#]	0.64[#]	1.05[#]
PP_5537	Membrane protein	2.65[#]	-1.40[#]	2.66[#]
PP_5718	Membrane protein	0.65[#]	0.93[#]	1.25[#]
PP_2966	Conserved membrane protein	-0.05[#]	0.43[#]	2.16[#]
PP_4023	Conserved membrane protein	1.10[#]	0.99[#]	0.94[#]
PP_2244	putative membrane component	2.52	2.29	2.10
PP_5096	putative membrane component	0.96	1.04	0.95
PP_5743	putative membrane component	0.64[#]	1.02[#]	1.05[#]

[*]: Comparing with the control group without carbamazepine dosage

[#]: false discovery rate (FDR) > 0.05

Table S15. Proteins relevant to cell membrane in recipient bacteria *P. putida* KT2440 after exposure of carbamazepine

**Protein**	**Protein description**	**Log_2(Fold Change of Protein Abundance)***		
		**Low-dosage**	**Medium-dosage**	**High-dosage**
OmpA	OmpA family lipoprotein	-0.25	0.03	0.87
OprG	outer membrane protein OprG	1.64	1.41	0.37
OprL	Peptidoglycan-associated lipoprotein	-1.48	0.14	1.77
TtgC	Probable efflux pump outer membrane protein TtgC	0.05	0.40	0.09
NP_742435.1	outer-membrane porin D	1.05	0.74	0.29
NP_742402.1	outer-membrane porin E	0.55	0.17	-0.31
NP_743345.1	outer membrane protein H1	1.36	0.67	-0.51
NP_745593.1	multidrug RND transporter membrane fusion protein	0.36	-0.15	0.81
WP_010954246.1	outer membrane protein assembly factor BamA	0.37	0.42	-0.20

*: Comparing with the control group without carbamazepine dosage

Table S16. Genes relevant to pilus generation in donor bacteria *E. coli* K-12 LE392 after exposure of carbamazepine

**Gene**	**COG Annotation**	**Log$_2$(Fold Change of FPKM)[*]**		
		**Low-dosage**	**Medium-dosage**	**High-dosage**
fimB	Replication, recombination and repair; Mobilome: prophages, transposons	0.22[#]	1.30[#]	0.49[#]
fimF	Pilin (type 1 fimbria component protein)	1.03[#]	2.46[#]	1.19[#]
fimG	Cell motility	-0.64[#]	0.19[#]	1.57[#]
fimH	Pilin (type 1 fimbria component protein)	-0.31[#]	1.41[#]	0.53
yagI	DNA-binding transcriptional regulator, IclR family	2.61	2.23	2.21

[*]: Comparing with the control group without carbamazepine dosage

[#]: false discovery rate (FDR) > 0.05

Table S17. Genes relevant to pilus production and transfer regulation in RP4 plasmid after exposure of carbamazepine

**Gene**	**COG Annotation**	**Log$_2$(Fold Change of FPKM)[*]**		
		**Low-dosage**	**Medium-dosage**	**High-dosage**
traA		0.99	0.81	0.84
traB		1.49	1.15	1.35

**Gene**	**COG Annotation**	**Log$_2$(Fold Change of FPKM)***		
		**Low-dosage**	**Medium-dosage**	**High-dosage**
traH		2.05#	1.80#	3.10#
traL		0.87	1.01	0.70
traP		2.16	1.63#	1.35#
traC1		1.18	0.78	0.70
traC2		1.51#	1.37#	1.61#
traI		1.14#	0.87#	0.77#
traJ		-0.31#	0.55#	1.06#
traM		0.73#	1.22	0.66#
trbA		-0.05#	0.18#	0.36#
trbB		0.09#	0.14#	-0.06#
trfA		0.42	0.26#	0.51
korA		-0.52#	-0.92#	-0.26#

*: Comparing with the control group without carbamazepine dosage

#: false discovery rate (FDR) > 0.05

References in Supporting Information

1 Zwietering, M. H., Jongenburger, I., Rombouts, F. M., & Van't Riet, K. (1990). Modeling of the bacterial growth curve. Applied and environmental microbiology, 56(6), 1875-1881.

2 Zhang Y, Gu AZ, He M, Li D, Chen JM. Subinhibitory concentrations of disinfectants promote the horizontal transfer of multidrug resistance genes within and across genera. *Environ Sci Technol.* 2017; 51: 570-580.

3 Zhang Z, Schwartz S, Wagner L, Miller W. A greedy algorithm for aligning DNA sequences. *J Comput Biol.* 2000; 7: 203-214.

4 Gao SH, Fan L, Peng L, Guo JH, Agullo-Barcelo M, Yuan ZG, *et al.* Determining multiple responses of pseudomonas aeruginosa pao1 to an antimicrobial agent, free nitrous acid. *Environ Sci Technol.* 2016; 50: 5305-5312.
